# Isolated Splenic Metastasis With Perisplenic Artery Lymph Node Involvement More Than 10 Years After Curative Resection of Ascending Colon Cancer: A Case Report

**DOI:** 10.7759/cureus.100737

**Published:** 2026-01-04

**Authors:** Ryuhei Noda, Naoki Kataoka

**Affiliations:** 1 Department of Surgery, Kishiwada Tokushukai Hospital, Kishiwada, JPN

**Keywords:** colorectal cancer, isolated metastasis, late recurrence, splenectomy, splenic metastasis

## Abstract

Splenic metastasis from colorectal cancer (CRC) is extremely rare, particularly as an isolated lesion, and most recurrences occur within five years. The presence of concurrent perisplenic artery lymph node metastases is unusual. A 71-year-old male who underwent right hemicolectomy for ascending colon adenocarcinoma (pT4aN0M0, Stage II) presented with elevated CA19-9 levels and a splenic tumor 10 years and 5 months after surgery. Imaging revealed a 9 × 8 cm splenic mass with suspected gastric wall invasion and swelling of the lymph node along the splenic artery. Positron emission tomography/computed tomography (PET/CT) confirmed high uptake in both lesions, and endoscopic ultrasound-guided fine-needle aspiration diagnosed adenocarcinoma. An open splenectomy with en bloc resection of the invaded gastric wall and No. 11p lymphadenectomy was performed. Pathology revealed a moderately differentiated adenocarcinoma in the spleen with gastric invasion and metastasis in the No. 11p lymph node. The patient recovered well after the surgery and completed adjuvant capecitabine chemotherapy. The patient remained recurrence-free six months postoperatively.

This case highlights the exceptional rarity of isolated splenic metastasis appearing after more than 10 years of disease-free status, accompanied by perisplenic artery lymph node involvement. Splenic metastasis is believed to be prevented by anatomical and immunological mechanisms; however, splenectomy has demonstrated survival benefits. Concomitant lymph node metastasis suggests a potential lymphatic route of spread, and this postulated mechanism is supported by recent multicenter mapping of splenic flexural colon cancers. Splenic metastasis may occur even after long disease-free intervals, and splenectomy with lymphadenectomy can be both therapeutic and informative. Therefore, vigilance among long-term CRC survivors is warranted.

## Introduction

Colorectal cancer (CRC) commonly metastasizes to the liver and lungs, whereas splenic metastases are rare. The spleen is generally considered an unfavorable site for tumor implantation due to several unique factors, including its rhythmic contractile nature, the sharp angle of the splenic artery, which may hinder hematogenous seeding, and the high concentration of immune cells within its reticuloendothelial system [1,2]. In autopsy studies, splenic metastases have been observed in only 4.4% of cases, with 1.6% originating from CRC [1]. Moreover, most splenic metastases occur as part of systemic dissemination, making solitary involvement particularly unusual [2,3]. Clinically, the frequency of isolated splenic metastasis is considered exceptional, with fewer than 100 cases reported worldwide as of 2012 [4].

Regarding the timing of recurrence, CRC recurrence usually occurs within five years of resection, reflecting the standard surveillance window based on established clinical guidelines. Late recurrence beyond 10 years is extremely rare, often challenging the traditional understanding of “cure” in CRC patients. In this report, we present an extremely rare case of isolated splenic metastasis occurring 10 years and 5 months after the curative resection of ascending colon cancer, accompanied by perisplenic artery (No. 11p) lymph node metastasis.

## Case presentation

A 71-year-old male was referred to our hospital for evaluation of a splenic mass, which was incidentally discovered at a local clinic, with an elevated CA19-9 level (253 U/mL). He had undergone right hemicolectomy 10 years and 5 months previously for ascending colon adenocarcinoma (tub2, pT4aN0M0, stage II). The patient remained disease-free for five years, after which surveillance was discontinued. His medical history included hypertension, dyslipidemia, and hyperuricemia.

**Figure 1:**
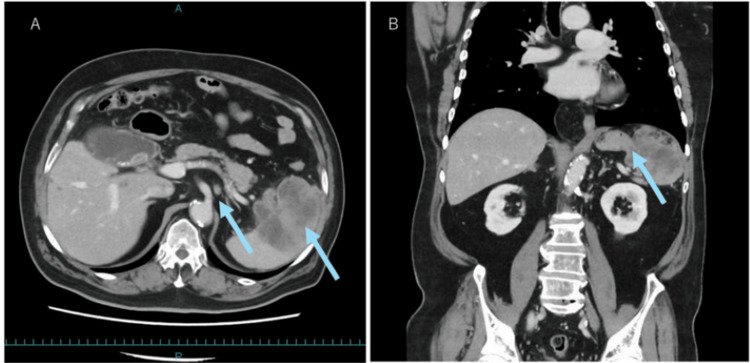
Contrast-enhanced abdominal computed tomography images (A) The coronal view shows a large, heterogeneously enhanced mass in the spleen (arrows). (B) The axial view demonstrates the 9 × 8 cm splenic tumor with suspected gastric wall invasion and a swollen lymph node adjacent to the celiac artery (No. 11p) (arrow).

On admission, a physical examination revealed a flat, soft abdomen with a healed midline scar. Laboratory findings showed preserved hepatic and renal function. Carcinoembryonic antigen levels were mildly elevated at 7.5 ng/mL. Contrast-enhanced computed tomography (CT) imaging revealed a 9 × 8 cm splenic mass with suspected gastric wall invasion and swelling of the lymph node to the left of the celiac artery (Figures 1A, 1B).

**Figure 2:**
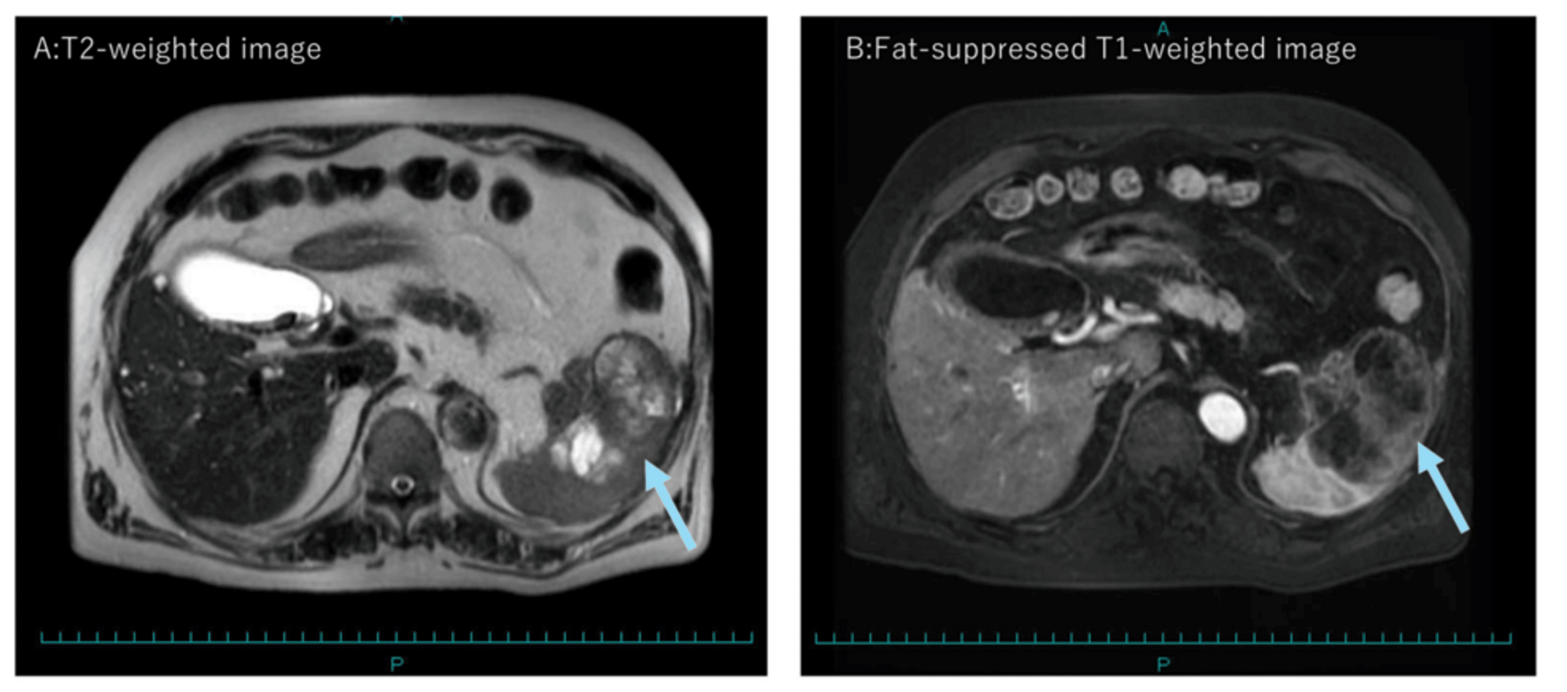
Magnetic resonance imaging of the spleen (A) The T2-weighted image shows a large mass with heterogeneous high signal intensity (arrow). (B) The fat-suppressed, T1-weighted dynamic contrast-enhanced image reveals irregular enhancement within the tumor (arrow).

**Figure 3:**
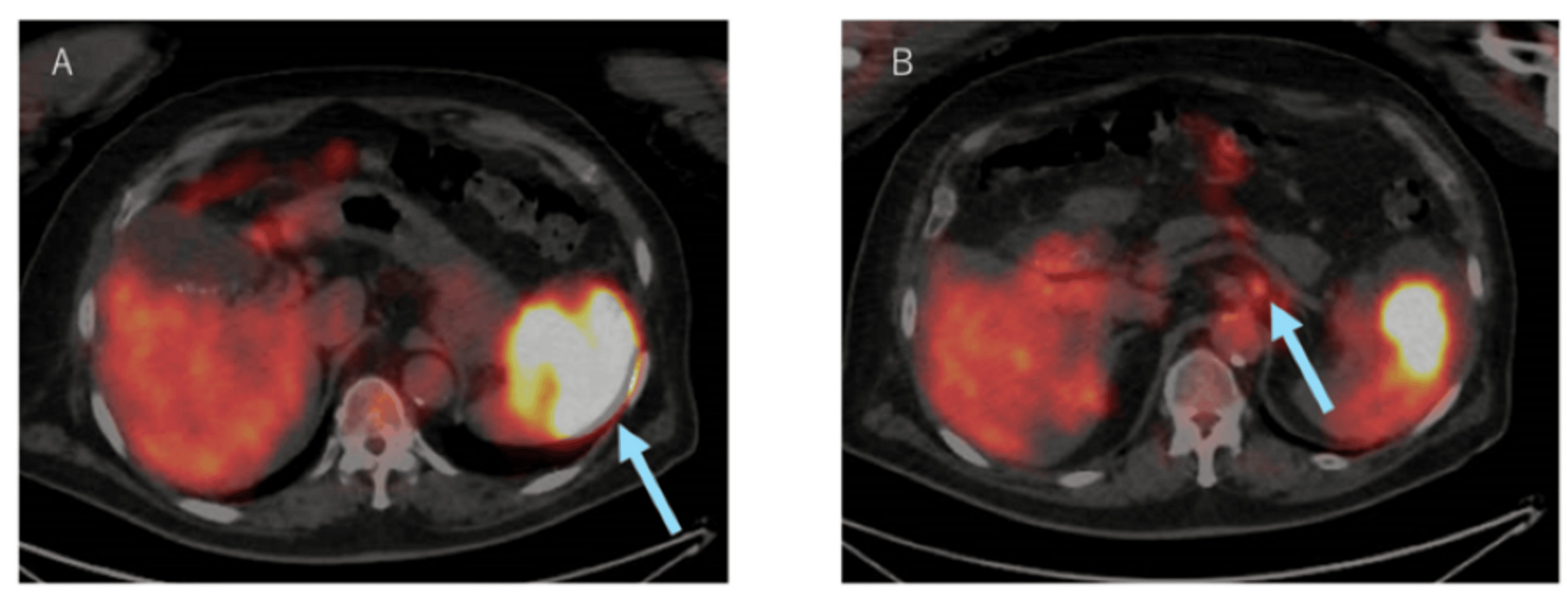
Positron emission tomography/computed tomography (PET/CT) Fused PET/CT images show intense fluorodeoxyglucose uptake in both the splenic tumor (A) and the perisplenic artery lymph node (B). The maximum standardized uptake values were 20.4 and 3.7, respectively.

Magnetic resonance imaging (MRI) confirmed an irregularly enhancing lesion (Figures 2A, 2B), while positron emission tomography/computed tomography (PET/CT) demonstrated increased uptake in both the splenic tumor (maximum standardized uptake value (SUVmax) 20.4) (Figure 3A) and the paraceliac lymph node (SUVmax 3.7) (Figure 3B). 

Endoscopic ultrasonography revealed heterogeneous vascularity, and endoscopic ultrasound-guided fine-needle aspiration of the splenic lesion confirmed the presence of adenocarcinoma. No other primary lesions were observed. Open splenectomy with en bloc resection of the invaded gastric wall and left diaphragmatic peritoneum was performed. Lymphadenectomy included the No. 11p node. Grossly, the spleen contained a 90 × 83 mm tumor with central necrosis. Microscopically, moderately differentiated adenocarcinoma was confirmed by invasion of the gastric wall. The No. 11p lymph node tested positive for metastases.

**Figure 4:**
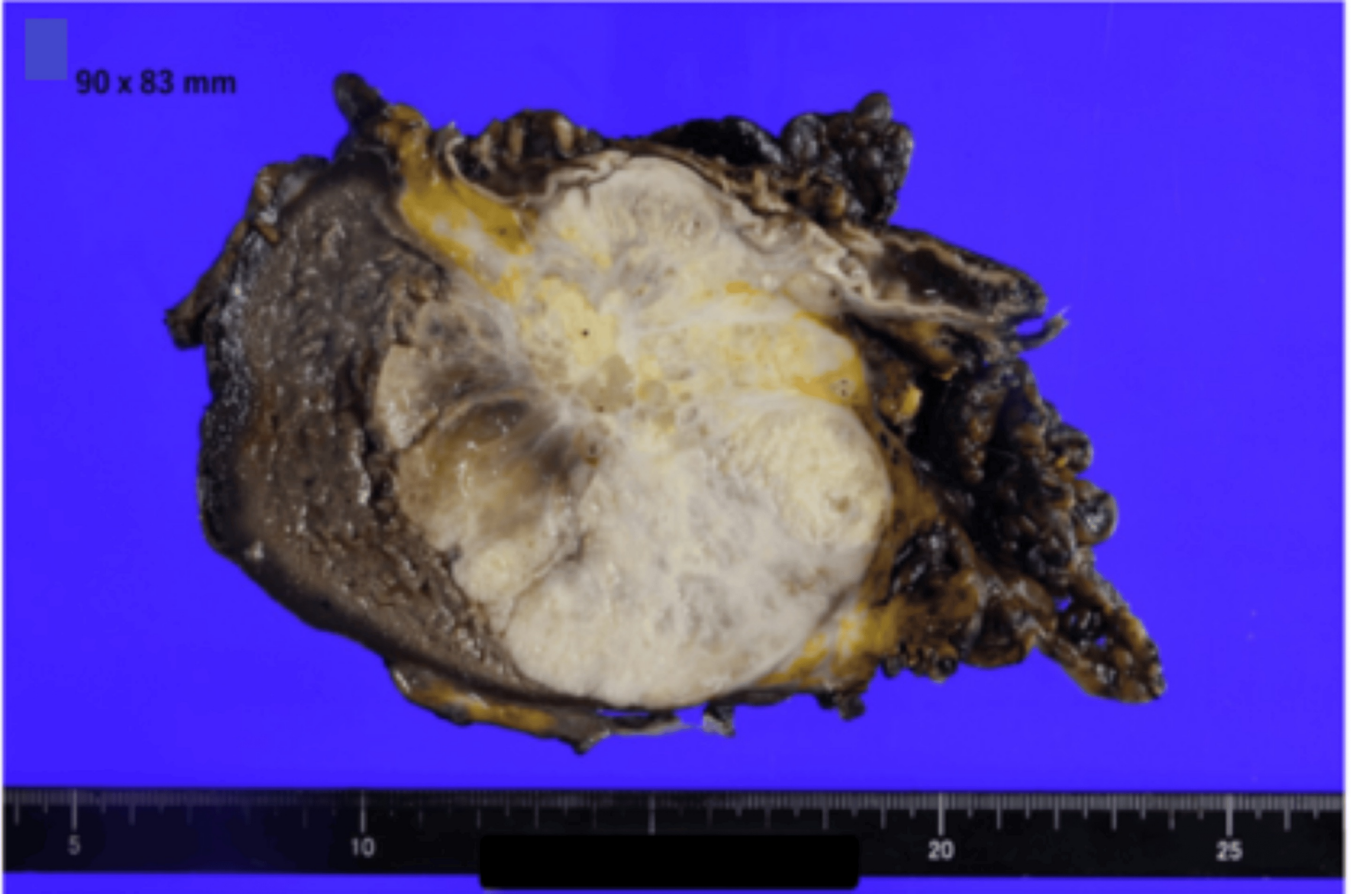
Gross appearance of the resected specimen The cut surface of the splenectomy specimen reveals a large, well-demarcated, whitish, solid tumor measuring 90 × 83 mm, with extensive central necrosis and cystic changes.

**Figure 5:**
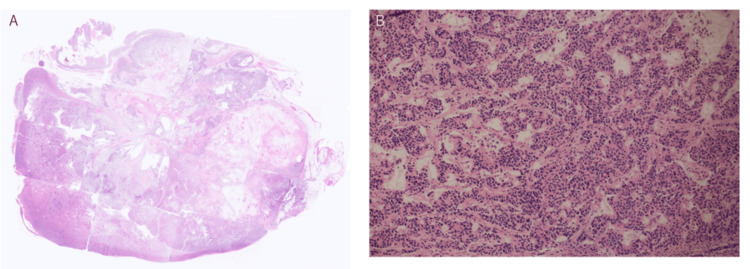
Histopathological findings (hematoxylin and eosin stain) (A) Low-power magnification (loupe view) shows a well-circumscribed carcinomatous nodule with central necrosis. (B) High-power magnification reveals tumor cells forming irregular glandular and cribriform structures, consistent with moderately differentiated adenocarcinoma. The tumor cells have enlarged nuclei, prominent nucleoli, and moderate atypia.

The splenic tumor exhibited extensive central necrosis (Figure 4). At the periphery, the tumor was predominantly composed of glandular structures with a focal cribriform architecture (Figure 5A). The tumor cells had enlarged nuclei, prominent nucleoli, and moderate nuclear atypia. Nuclear polarity was irregular, and scattered tumor cells demonstrated mucin production (Figure 5B). Overall, these findings were consistent with those of a moderately differentiated adenocarcinoma. Histological features closely resembled those of the patient’s previous ascending colon carcinoma, supporting the diagnosis of splenic metastasis from colorectal adenocarcinoma.

The postoperative course was complicated by an intra-abdominal abscess requiring drainage; however, the patient recovered and was discharged on postoperative day 22. The patient completed eight cycles of adjuvant capecitabine and remains recurrence-free six months postoperatively.

## Discussion

This case represents an exceptionally rare occurrence of isolated splenic metastasis from CRC more than 10 years after curative resection, accompanied by perisplenic artery (No. 11p) lymph node involvement.

Splenic metastases are uncommon in CRC and most often occur with systemic dissemination. Berge reported splenic metastasis in 4.4% of 7,165 cadavers, with only 1.6% being colon cancer [1]. Clinically, fewer than 100 cases of isolated splenic metastases from CRC were reported worldwide until 2012 [2]. Most reported splenic metastases occur with liver or lung involvement, and isolated lesions are exceedingly rare [3,4].

The concomitant perisplenic artery lymph node metastasis in our case makes this even more unusual. Efared et al. reported splenic metastasis associated with splenic hilar lymph node involvement [5]; however, metastasis to the 11p nodes has rarely been documented. Recent multicenter mapping data demonstrated that splenic flexural colon cancers rarely metastasize to the splenic artery nodes (0.7%), supporting the biological plausibility of our case [6].

Two possible metastatic routes may be considered: (1) lymphatic spread from the primary site via the celiac/splenic artery nodes before reaching the spleen, or (2) hematogenous metastasis to the spleen followed by secondary lymphatic dissemination. Many cases, including ours, have shown lymphatic invasion by the primary tumor, suggesting that lymphatic routes should be reconsidered in the pathogenesis of splenic metastasis [5].

Resistance to metastasis in the spleen has been attributed to anatomical and physiological factors, including the sharp angle of the splenic artery, poor afferent lymphatic drainage, rhythmic contractions expelling tumor cells, and reticuloendothelial immune functions [1,3]. However, surgical resection provides significant survival benefits when metastasis occurs.

Fujita et al. reported improved outcomes for isolated splenic metastasis compared with non-isolated disease, with long-term survival beyond nine years [7]. Tivadar et al. reviewed 83 patients and reported a median overall survival of 84 months after splenectomy, reinforcing its therapeutic role [8]. Notably, Rizzo et al. [9] documented favorable three- and five-year survival rates (82.3% and 68.8%, respectively) after splenectomy for isolated splenic metastases.

Building on these favorable outcomes, Table 1 summarizes reported cases of isolated splenic metastasis occurring five years or more after primary surgery. This includes the cases of Sileri et al. [10] and Jain et al. [11], who reported recurrence intervals of five and seven years, respectively, with both achieving no evidence of disease (NED) at 12 months post-splenectomy. Our present case is especially remarkable, with an exceptionally long interval of 10 years and 5 months. Despite the presence of a splenic artery lymph node metastasis (No. 11p), the patient remains NED at six-month follow-up. These findings underscore the clinical significance of aggressive surgical intervention for isolated splenic recurrence, even a decade after the initial operation.

**Table 1:** Reported cases of isolated splenic metastasis from colorectal cancer occurring more than five years after primary surgery LN, lymph node; NED, no evidence of disease

Author (year)	Primary site	Interval to splenic metastasis	LN involvement	Treatment	Outcome
Sileri et al. (2009) [10]	Sigmoid colon	5 years	None	Splenectomy	NED at 12 months
Jain et al. (2011) [11]	Rectum	7 years	None	Splenectomy	NED at 24 months
Present case	Ascending colon	10 years 5 months	No. 11p (+)	Splenectomy + LN dissection	NED at 6 months

Regarding the postoperative management, capecitabine was selected as the adjuvant chemotherapy regimen. A key factor in this decision was the patient’s clinical history: following the initial surgery for the primary tumor, the patient had completed adjuvant capecitabine monotherapy and remained disease-free for over a decade. This long-term remission suggested that the tumor was chemosensitive to fluoropyrimidines. Furthermore, considering the patient’s age (71 years) and the desire to maintain quality of life by avoiding the cumulative neurotoxicity associated with oxaliplatin, we concluded that re-challenging with capecitabine was a balanced and rational therapeutic approach.

In summary, this case highlights three key points: (1) isolated splenic metastasis can occur even after a prolonged disease-free interval of >10 years; (2) concomitant perisplenic artery lymph node metastasis suggests possible lymphatic spread, consistent with recent mapping studies; and (3) splenectomy with lymphadenectomy offers both therapeutic benefit and pathological insight in selected patients.

## Conclusions

We report a rare case of isolated splenic metastasis, with perisplenic artery lymph node involvement, that occurred >10 years after curative resection of ascending colon cancer. This case highlights the importance of long-term vigilance in CRC survivors and supports the use of splenectomy with lymphadenectomy as a therapeutic option.
